# Impact of Brazilian papers in cardiology and cardiovascular sciences in the last decade

**DOI:** 10.5935/abc.20170005

**Published:** 2017-01

**Authors:** Luiz Felipe P. Moreira

**Affiliations:** Instituto do Coração, Hospital das Clínicas, Faculdade de Medicina - Universidade de São Paulo - USP, São Paulo, SP - Brazil

**Keywords:** Cardiovascular Diseases, Cardiology, Periodicals as Topic, Bibliometrics

During the last decade, there has been a significant increase in the number of Brazilian publications in cardiology and cardiovascular sciences in important international citation indexing platforms. This occurred in Brazil and in most Latin American countries at approximately 13% per year between 1999 and 2008 according to the study by Colantionio et al.^1^ This represents nearly 3% of all articles published in international journals indexed in the Web of Science platform, maintained by Thompson-Reuters, and Scopus-Scimago, provided by Elsevier.

Although most of our indexed articles have been published in international journals, the citation indexes achieved by Brazilian and Latin American authors are usually lower than those from countries with higher income or higher Human Development Index.^1^ This is even more evident for studies conducted in national institutions in comparison with those developed with some degree of international cooperation.

**Figure 1:**
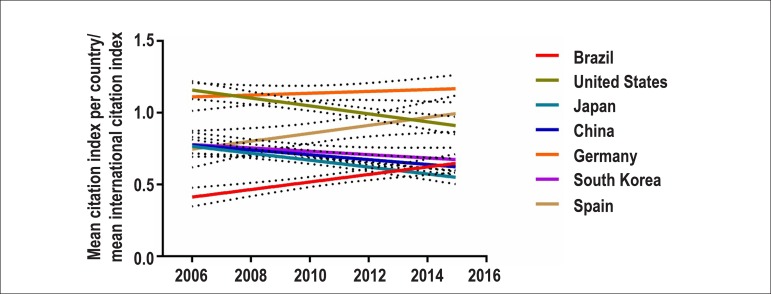
Relationship between the mean citation indexes of papers published in the fields of cardiology and cardiovascular sciences in each country and the mean international citation index in the period from 2006 to 2016.

As compared with international citations, using the data from Scimago country ranking,^2^ the mean citation index of Brazilian papers in cardiology has progressively increased from less than 0.5 to nearly 0.65 in the last ten years (Figure 1). Today, this index is similar to that of countries like Japan, South Korea and China.

Advances in the quality of Brazilian papers in cardiology has occurred along with the improvement of Brazilian researchers' qualification thanks to Master and Doctoral degree programs and incentive programs for scientific research supported by agencies and national medical societies. On the other hand, approximately 35% of Brazilian papers in cardiology or cardiovascular sciences have been published in *Arquivos Brasileiros de Cardiology* or other Brazilian journals, and less than 50% of papers in journals with an impact factor greater than 1.6.

Previously, we have reported that the citation indexes of studies on major subjects in cardiovascular sciences, such as 'myocardial revascularization' and 'atrial fibrillation' published in Brazilian journals are not different from those of articles published in other countries.^3^ Aiming to expand this analysis, we assessed 968 articles in cardiology indexed in the Web of Science, written exclusively by Brazilian authors and published between 2010 and 2014.

**Figure 2:**
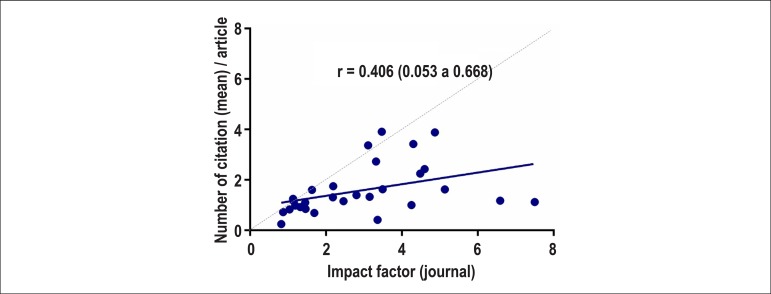
Correlation between mean number of citations obtained from 968 articles of Brazilian authors published in 30 journals in cardiology and cardiovascular sciences within 2 years of their publication and the impact factor in which these articles were published.

We considered the mean citation index of the 30 journals with the highest number of articles published, and assessed correlations between the number of times these articles were cited and the impact factor of the journals in which these articles were published. There was a weak correlation between the mean number of times these papers were cited within two years of publication and the impact factor of the journals, especially in higher impact journals (Figure 2).

These findings confirm that, despite recent advances, the citation indexes obtained from Brazilian authors are still lower than the mean international index. Besides, they highlight the importance to analyze the citation indexes of each study. These are currently available in the main international citation indexing systems that, in turn, provide an online, daily update of these parameters. For this reason, the 'value' of a publication may not be related to the journal impact. In fact, the citation index of scientific publications has been used by most of Brazilian fostering agencies and the Lattes Platform, a database of Brazilian researchers' resumes.

On the other hand, one of the main ranking criteria of graduate programs in Brazil, used by the Coordination for the Improvement of Higher Education Personnel (CAPES) of the Ministry of Health, is based on Qualis system. Qualis classifies scientific production of graduate programs according to the impact factors of journals in which the papers are published, without taking into consideration the indexes of each publication. Thus, a revision of this method is required to promote adequate fostering of research and incentive for the publication of Brazilian papers in internationally indexed journals.

A positive attitude towards the progress of scientific research in Brazil also depends on the maintenance of government and private funding to scientific research, on the expansion of training programs on clinical and laboratory studies, and improvement of Master's and Doctoral programs. Besides, the development of clinical trials and multicenter or multinational studies on major cardiovascular diseases by our centers and medical societies^4,5^ represent important initiatives of great impact and an adaptation of scientific knowledge to national conditions.
